# Diversity of Bacterial Community in the Oxygen Minimum Zones of Arabian Sea and Bay of Bengal as Deduced by Illumina Sequencing

**DOI:** 10.3389/fmicb.2019.03153

**Published:** 2020-01-21

**Authors:** Genevieve L. Fernandes, Belle Damodara Shenoy, Samir R. Damare

**Affiliations:** ^1^Biological Oceanography Division, CSIR-National Institute of Oceanography, Dona Paula, India; ^2^Department of Microbiology, Goa University, Taleigao, India; ^3^CSIR- National Institute of Oceanography Regional Centre, Visakhapatnam, India

**Keywords:** canonical correspondence analysis, functional traits, high-throughput sequencing, Indian Ocean, oxygen minima

## Abstract

The Indian Ocean harbors oxygen minimum zones (OMZs) in the Arabian Sea and Bay of Bengal, with dissolved oxygen < 20 μM, located at the mid-depths of the water column. Till date, high-throughput sequence-data on depth-wise distribution of prokaryotic communities have rarely been reported from these OMZs. The present study aimed to characterize the prokaryotic diversity inhabiting Arabian Sea Time Series (ASTS) and India’s Idea 2 (II2) in the Arabian Sea, and Bay of Bengal Time Series (BoBTS) in the Bay of Bengal OMZs based on amplicon sequencing of 16S rRNA gene regions, along six sampled depths in the water column. High prokaryotic richness was observed in the Arabian Sea and Bay of Bengal samples. Operational taxonomic units (OTUs) in the range of 1249–3298 were identified, wherein, less prokaryotic diversity was observed at surface and within oxygen minimum depths. At phylum level, most OTUs were affiliated to Bacteroidetes, Chloroflexi, Cyanobacteria, Marinimicrobia, Planctomycetes, and Proteobacteria. Prokaryotic community differed between ASTS, II2 and BoBTS locations along varying physicochemical conditions. Predictive functional profiling of the bacterial communities suggested the involvement of abundant microbes in nitrogen and sulfur metabolism pathways. Bacterial isolates belonging to genera from the clades, δ-Proteobacteria and γ-Proteobacteria, described previously for their participation in biogeochemical cycling of N-and-S in the OMZs were reported from deoxygenated waters of both the basins. Bacteria involved in anammox such as *Candidatus* Scalindua were found to be relatively high at ASTS and II2 locations in the Arabian Sea. Further studies are required to ascertain the role of abundant bacteria along the dynamic oceanographic processes in the OMZs.

## Introduction

Oxygen minimum zones (OMZs), also termed as shadow zones, are regions where oxygen saturation in seawater is persistently low, i.e., below 20 μM (0.5 ml/L) at intermediate depths of the water column (∼100 to 1300 m) (Levin, 2003; Gilly et al., 2013). The vast ocean harbors four known permanent tropical OMZs in the Pacific and Indian Ocean: Eastern North Pacific (ENP) in the north, Eastern South Pacific (ESP) in the south; Arabian Sea (AS) in the west and Bay of Bengal (BoB) in the east. OMZs are hypothesized to have formed due to the biological processes that reduce the oxygen concentration along with physical processes that prevent these waters from evenly mixing with the surrounding water (Paulmier and Ruiz-Pino, 2009). The Indian Ocean, including Arabian Sea and Bay of Bengal, covers more than half (59%) of the area of the OMZs in comparison to the other world oceans. The AS and BoB basins are intense due to common geographical characteristic of being landlocked toward the north, thus restricting the ventilation of the thermocline (Qasim, 1999; Naqvi, 2006). The AS-OMZ experiences intense low dissolved oxygen (DO) concentration of < 2 μM (0.05 ml/L) between 100 and 1000 m depth in the water column, while the BoB-OMZ has DO levels < 4 μM (0.1 ml/L) at mid depths in the water column. Although the difference in oxygen concentration between these two basins is just 2 μM, a large-scale denitrification and reducing of other electron acceptors takes place in AS, in contrast to BoB that inhibits denitrification rates by 50% (Naqvi, 2006; Johnson et al., 2019). The variation in the oxygen intensity between AS-OMZ and BoB-OMZ is suggested to be due to varying levels of primary productivity, differing intensities of mesoscale eddies and a contrasting amount of organic matter and oxygen that gets transported in these basins (McCreary et al., 2013). In fact, the BoB is believed to be less productive than the AS, much contributed to the freshwater input from the rivers, which not only reduces the salinity on the surface, but also induces stratification (Gomes et al., 2000; Madhupratap et al., 2003; Singh and Ramesh, 2015).

Marine OMZs harbor microbial communities that are known to play significant biochemical roles along a steep oxygen gradient in the water column (Galán et al., 2009). Microbial activities in the OMZs have shown to contribute 30–50% nitrogen loss in the ocean (Lam and Kuypers, 2011). Although the OMZs are well-studied regions for nitrogen cycling, recent studies, based on metagenomic and biochemical methods, from the Pacific and Indian Oceans investigated the role of microbes in sulfur cycle (Canfield et al., 2010; Menezes et al., 2018). Previous studies, based on culture-based and culture-independent techniques, from the AS and BoB focused on bacterial and archaeal communities that dominate the coastal and open ocean OMZ and non-OMZ regions. The pelagic microbial communities distributed in the Indian Ocean have been reported recurrently with the occurrence of Proteobacteria as the most dominant phyla. Culture-dependent and independent analysis of the AS and BoB OMZ and non-OMZ waters have shown a high representation of δ-Proteobacteria and γ-Proteobacteria affiliated to Proteobacteria (Riemann et al., 1999; Fuchs et al., 2005; Divya et al., 2011; Jain et al., 2014; Wang et al., 2016; Bristow et al., 2017; Mulla et al., 2017; Bandekar et al., 2018; Qian et al., 2018; Rajpathak et al., 2018; Fernandes et al., 2019; Paingankar et al., 2019). Other taxa at class-level including the Betaproteobacteria, Deltaproteobacteria, Actinobacteria, Nitrospinia, Planctomycetacia, and SAR406 clade were also well-reported however varied along the depths and sites sampled in the Indian Ocean OMZs (Fuchs et al., 2005; Bandekar et al., 2018; Fernandes et al., 2019). Pelagic nitrogen and sulfur cycling in the OMZs of the Indian Ocean have recently revealed presence of nitrate-reducing and sulfur-oxidizing bacteria based on biochemical of culture-dependent studies (Mulla et al., 2017; Menezes et al., 2018; Fernandes et al., 2019). Metagenomic analysis in the AS OMZ have revealed presence of denitrifiers and annamox bacteria (Bandekar et al., 2018). While diversity studies in the BoB OMZ revealed presence of Gammaproteobacterial sulfur oxidizers (GSO) belonging to the SUP05 clade and further functional gene analysis reviled nitrate reductase genes thus suggesting a role of sulfur and nitrogen cycling in the BoB OMZ (Bristow et al., 2017). The major groups of abundant taxa identified in this region are reported to harbor significant taxa at finer taxonomic levels which participate in biogeochemical processes that occur in the oxygen deficient waters (Ulloa et al., 2013). In order to tap these taxa, improved metagenomic techniques need to be employed, in getting greater sequence depth (Sogin et al., 2006; Kennedy et al., 2010; Shokralla et al., 2014) then prior employed cloning and Sanger sequencing methods. Microbial ecologists over the past few years have developed effective ways to characterize microbial communities. Application of high throughput sequencing technologies has made way to microbial community structure analysis using amplicon sequencing and metagenomics. These techniques can substantially improve our understanding of microbial diversity in a given environmental sample (Tringe and Hugenholtz, 2008; Caporaso et al., 2012; Lynch and Neufeld, 2015). However, due to limited studies from the AS and BoB OMZs on diversity, composition, and abundance of bacterial and archaeal communities based on amplicon sequencing of 16S rRNA gene regions there is a need to analyze these regions with modern metagenomic tools.

In the present study, bacterial diversity in the water column of the OMZs across three sampling locations in the northern Indian Ocean, viz. Arabian Sea time series (ASTS), India’s Idea 2 (II2) and Bay of Bengal time series (BoBTS) was investigated. The bacterial communities were characterized using Illumina sequencing of 16S ribosomal RNA gene regions from 18 water samples collected from the three locations. The distribution of bacterial communities in relation with physicochemical environmental variables and predicted functional gene composition from the sampled OMZ regions was also studied.

## Materials and Methods

### Sampling Locations and Methodology

Seawater samples were collected from three stations during two successive cruises onboard the Research Vessel Sindhu Sadhana (RV SSD) in the northern Indian Ocean. The BoBTS (Latitude: 18.002728°N, Longitude: 89.01749°E, water depth: 2230 m) located in the northeastern Bay of Bengal was sampled on 2^nd^ April 2016 during cruise #SSD020. The ASTS (Latitude: 16.997341°N, Longitude: 68.005768°E, water depth: 3535 m) and II2 (Latitude: 9.000113°N, Longitude: 67.999901°E, water depth: 4530 m) located in the Arabian Sea were sampled on 25^th^ and 30^th^ September 2016, respectively (Supplementary Figure S1). Seawater samples were collected using a Conductivity-Temperature-Depth (CTD) rosette system fitted with 24 Niskin bottle sampler (Seabird Electronics, Washington, NY, United States) mounted with an oxygen sensor. Ten liters seawater were filtered through 0.22 μm sterivex filters (Millipore, United States) using a peristaltic pump. The filters were filled with DNA storage buffer (50 mM Tris pH 8.3, 40 mM EDTA, and 0.75M sucrose), and stored at −80°C. Six depths were chosen according to the oxygen distribution at the sampled sites viz., surface, four depths within oxygen minima [upper interphase (upper OMZ), core OMZ (2 depths), lower interphase (lower OMZ)] and near bottom. Nitrate and Nitrite were measured from the frozen samples carried to the on-shore laboratory using Skalar Autoanalyser (Skalar Analytical, Netherlands) following standard methods (Grasshoff et al., 1983).

### DNA Extraction, Library Preparation, and Sequencing

The sterivex filter cartridge was cracked opened aseptically under a laminar flow hood using a clean plyer (cleaned with absolute ethanol 99.9%). The filter paper was shredded with a clean college plier (dipped in absolute ethanol 99.9%). Filter paper pieces were then added to the MoBio PowerWater bead tubes containing lysis buffer and manufacturer’s protocol was followed for the genomic DNA extraction. Six DNA samples from the BoBTS during the SSD020 cruise were vacuum dried in a vacuum concentrator (Eppendorf, Germany) and outsourced for sequencing. 16S rRNA gene libraries were prepared using 16S rRNA gene V3 region-specific targeting proprietary primers at Genotypic Technology, Pvt. Ltd., Bengaluru, India. Briefly, 50 ng of genomic DNA was used to amplify the 16S rRNA gene V3 hypervariable region for 26 cycles using KAPA HiFi Hot Start PCR Kit (Boston, MA, United States). The forward and reverse primer concentration was kept at 0.2 μM each. A positive control and non-template control samples were run to validate PCR. The amplicons were analyzed on 1.2% agarose gel (Round 1 PCR). 1 μl of 1:10 diluted round 1 PCR amplicons was used for Indexing PCR (Round 2). Here, the round 1 PCR amplicons were amplified for 8–10 cycles to add Illumina Sequencing barcoded adaptors (Nextera v2 Index Kit, Illumina, and United States). The Illumina Adapter Sequences were: 5′-AATGATACGGCGACCACCGAGATCTACAC [i5] TCGTC GGCAGCGTC and 5′-CAAGCAGAAGACGGCATACGAGAT [i7] GTCTCGTGGGCTCGG. Round 2 PCR amplicons were analyzed on 1.2% agarose gel. Similarly, 12 DNA samples from the cruise SSD026 were outsourced for sequencing to Eurofins Genomics India, Pvt. Ltd., Bengaluru, India. 50 ng of genomic DNA was amplified with 16S rRNA gene V3–V4 region-specific primers (F-GCCTACGGGNGGCWGCAG; R- ACTACHVGGGTATCTAATCC). A positive control and non-template control samples were run to validate PCR. The amplicons were analyzed on 1.2% agarose gel (Round 1 PCR). The amplicon libraries were prepared using the Nextera XT Index Kit (Illumina, Inc.) as per the 16S rRNA gene Metagenomic Sequencing Library preparation protocol (Part # 15044223 Rev. B). Amplification of the 16S rRNA gene targeting V3–V4 regions specific for bacteria was carried out (Round 2). Here, the round 1 PCR amplicons were amplified for 8–10 cycles to add Illumina Sequencing barcoded adaptors (NexteraXT v2 Index Kit, Illumina, and United States). Round 2 PCR amplicons were analyzed on 1.2% agarose gel. The amplicon library was purified by 1X AMPureXP beads and quantified using Qubit fluorometer. The amplified libraries were analyzed in 4200 Tape Station system (Agilent Technologies) using D1000 Screen tape as per manufactures instructions.

### Sequence Analysis

The Illumina paired-end reads for V3 (150^∗^2), and V3–V4 (2^∗^300) were demultiplexed using the bcl2fastq1 tool. The paired-end reads were quality checked using FastQC (Andrews, 2010). The raw reads having primer sequence and high-quality bases were selected and stitched using Fastq-join3, for further analysis using Quantitative Insights In Microbial Ecology (QIIME) pipeline (Caporaso et al., 2010). The sequences generated from each sample were clustered into operational taxonomic units (OTUs) using *uclust* module at 97% sequence similarity and each resulting cluster typically represents a species. The taxonomy of these clusters was assigned based at 97% sequence similarity against the SILVA database version 132 (Quast et al., 2013). The biom file generated was taken ahead for further advanced analysis and visualization. The 16S rRNA gene sequence-data was submitted to the National Center for Biotechnology Information (NCBI) under BioProject ID PRJNA508851.

### Statistical and Functional Analysis

QIIME program was used to calculate taxonomic richness (OTUs, Chao-1, Simpson and Shannon diversity). R studio V3.5.1 (R Core Team, 2015) was used to construct hierarchical clustering based on Euclidian similarity index and computed using gplots, heatmap.plus, and RColorBrewer packages (Day, 2012; Neuwirth, 2014). Beta diversity at class level was calculated between the three sites using non-metric multidimensional scaling analysis (NMDS) based on Bray–Curtis distance using Phyloseq package (McMurdie and Holmes, 2013). Bacterial community dynamics at generic level along with six environmental variables [Temperature (°C), dissolved oxygen (μM), nitrate (μM), nitrite (μM), salinity (PSU), and pH] were analyzed using canonical correspondence analysis (CCA) at ASTS, BoBTS and II2 sampled locations using Past-3 software V3.23 (Hammer et al., 2001).

The predictive functional assignments of the bacterial communities of the 18 samples were obtained using the Silva-Tax4Fun (Aßhauer et al., 2015). The SILVA-labeled OTU tables were used as an input file in Tax4Fun, which is an open-source R package. In Tax4Fun the SILVA-labeled OTUs is converted into prokaryotic KEGG organisms which is further normalized by 16S rRNA copy number obtained from the NCBI genome annotation (Kaiser et al., 2016). The bacterial communities were assigned their predictive functions by linearly combining the normalized taxonomic abundances into the precomputed association matrix of KEGG Ortholog reference profiles to Silva defined microorganisms constructed by Tax4Fun. Differences in functional gene composition groups among the depths at three stations were tested with Tukey–Kramer test using statistical analysis of amplicon sequences (STAMP V2.1.3).

## Results

### Hydrographic Physicochemical Parameters

**FIGURE 1:**
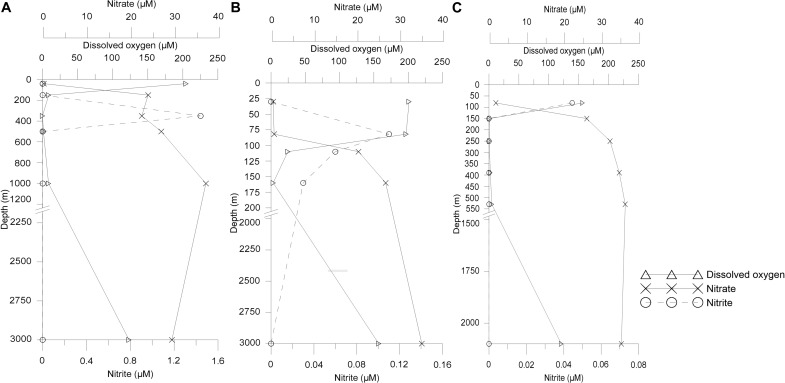
Vertical distribution of concentrations of dissolved oxygen (DO), nitrate (NO_3_), and nitrite (NO_2_) from sampled depths at **(A)** Arabian Sea time series (ASTS), **(B)** India’s Idea 2 (II2), and **(C)** BoBTS.

The temperature decreased as the depth increased, while higher salinity was observed at surface sampled depth of ASTS (36.9 PSU) and II2 (36.5 PSU) than BoBTS (34.1 PSU). Dissolved oxygen (DO) profile of ASTS and BoBTS is typical of an OMZ, i.e., the surface and near bottom depths are well-oxygenated. The mid four depths showed low oxygen concentration (Table 1) and, with depths of 350 and 250 m showing the lowest DO at ASTS and BoBTS, respectively, while the DO at II2 was above the defined OMZ concentration (>20 μM) at all sampled depths except 160 m (3.2 μM). The depth profile of dissolved oxygen (DO), nitrite (NO_2_), and nitrate (NO_3_) at ASTS, II2, and BoBTS is shown in Figure 1. Surface depths at all three stations exhibited low nitrate concentration, while it was observed in the OMZ and near bottom depths. The nitrite accumulation was evident only at 350 m (1.44 μM) at ASTS, while II2 showed low concentration within 82–201 m depths. However, at BoBTS nitrite was below detectable limits along the depths sampled except the surface depth (81 m).

**TABLE 1:** Tabulation of data on physical factors along with prokaryotic diversity estimates (at 97% similarity) for water collected from II2, ASTS, and BoBTS using CTD.

**Sampling station**	**Water depth (m)**	**Dissolved oxygen (μM)**	**Temperature (°C)**	**pH**	**Salinity (PSU)**	**OTUs**	**Chao-1**	**Shannon (H)**	**Simpson_1-D**
**II2**	30	201	28.5	8.08	36.5	1249	1724	5.92	0.951
	82	197	28	8.07	36.6	1692	2263	6.87	0.974
	110	24.6	23.7	7.71	36	1560	2030	7.01	0.980
	160	3.17	17.5	7.52	35.5	1427	1884	7.02	0.984
	201	34.5	15	7.55	35.3	1457	1821	7.24	0.985
	3000	157	1.8	7.58	34.7	1808	2470	7.20	0.974
**ASTS**	40	204	28	8.08	36.9	1347	1841	5.97	0.956
	150	8.84	18.6	7.58	35.7	1792	2367	7.48	0.985
	350	0	13.7	7.48	35.7	1131	1517	6.30	0.971
	500	1.34	12.2	7.49	35.6	1328	1662	6.14	0.919
	1000	8.57	8.5	7.44	35.3	1646	2187	6.70	0.954
	3000	123	1.8	7.56	34.7	1848	2303	6.75	0.968
**BoBTS**	81	156	27.8	7.85	34.1	1881	2638	5.78	0.952
	150	1.21	17.3	7.53	34.9	3233	4712	7.06	0.975
	250	0.62	12.5	7.45	35	1816	2504	5.88	0.957
	390	2.23	10.9	7.44	35	2256	3245	6.00	0.951
	530	4.82	9.8	7.44	35	2720	3640	6.67	0.969
	2100	121	2.4	7.82	34.8	3298	4455	6.71	0.972

### Alpha Diversity of Bacterial Communities

There were 1249–3298 OTUs identified in the 18 libraries, and the number of OTUs varied with the DO concentration (Table 1). The rarefaction curves (at 97% cutoff value) were bent toward the saturation plateau, indicating that the sampling sizes were sufficient (Supplementary Figure S2). The Chao-1, Shannon, and Simpson indices ranged from 1517–4712, 5.92–7.48, and 0.919–0.985, respectively. Moreover, OTUs, Chao-1 and Shannon diversity of all three stations were low at sampled surface depths (II2- 30 m, ASTS- 40 m, BoBTS- 81 m) and in the core OMZ depths at minimum DO concentrations (II2- 160 m, ASTS- 350 m, BoBTS- 250 m) (Table 1).

### Prokaryotic Community Structure

**FIGURE 2:**
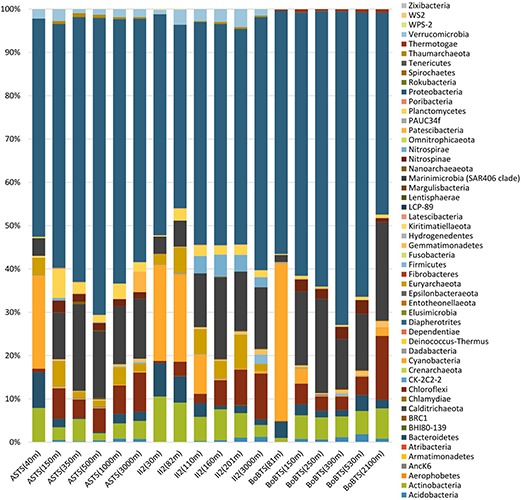
Percentages of relative abundance (x-axis) and depth-wise representation of bacterial phyla (y-axis) at the Arabian Sea time series (ASTS) and India’s Idea 2 (II2) in the Arabian Sea and Bay of Bengal time series (BoBTS) in the Bay of Bengal.

Fifty-two bacterial phyla were identified in the sampled sites (Figure 2). Proteobacteria dominated in all the samples (accounting for 42.35–68.57%). Cyanobacteria and Bacteroidetes were more prominent at the surface depths and decreased drastically to less than 1% at the OMZ and near bottom depths in all samples. In contrast, Marinimicrobia (clade SAR406 Marine Group A) and Chloroflexi were prominent at the deeper depths, while Actinobacteria was evenly distributed across the depths sampled. The phylum Planctomycetes was higher at ASTS and II2 sampling sites in comparison to BoBTS.

**FIGURE 3:**
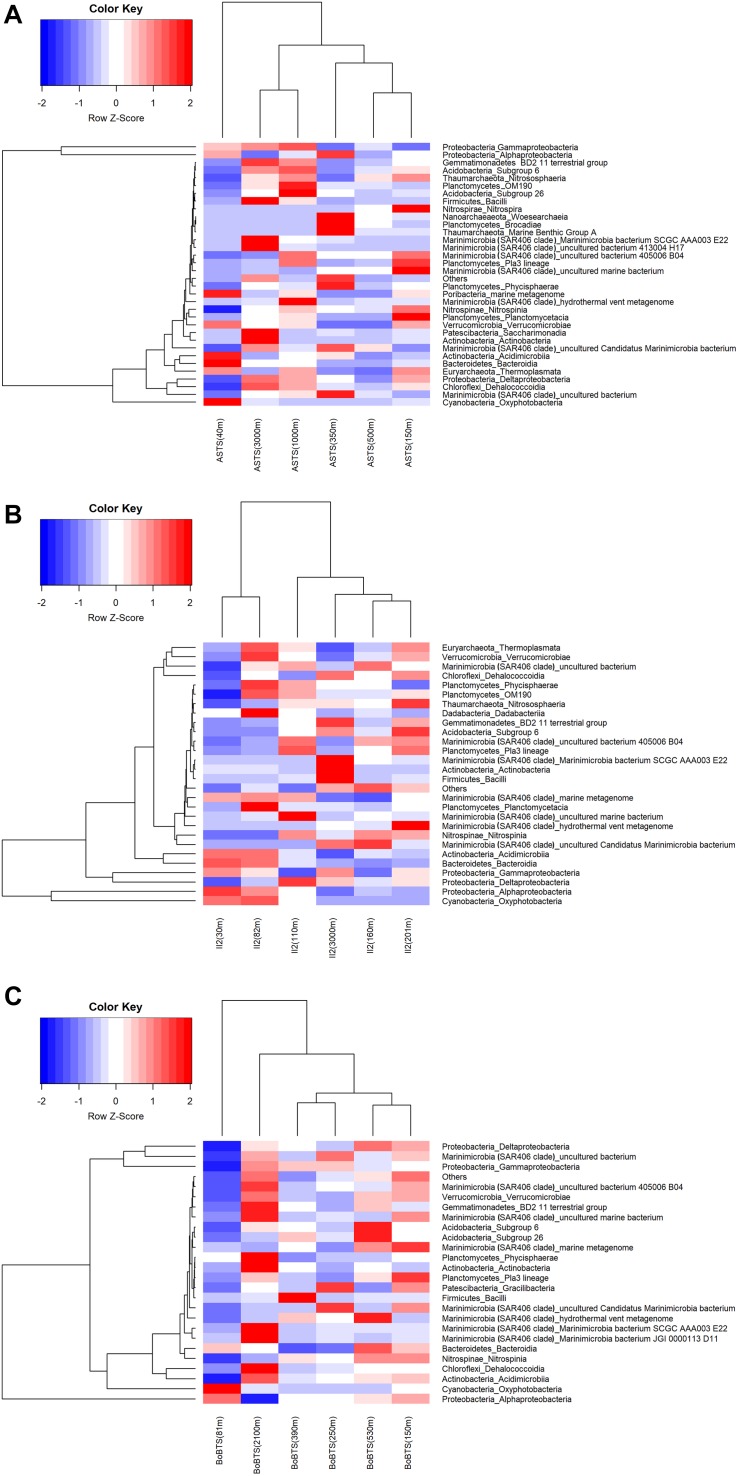
Heatmap generated using the relative abundance of bacteria at phyla_class level. The x-axis represents the distribution of bacterial communities in the sampled depths and y-axis represents the class-level taxonomy. The heatmap scale displays the row Z score (Z score = [actual relative abundance of a bacterial taxa at class-level in a specific depth of the water column – mean relative abundance of the same taxa along the sampled depths in the water column]/standard deviation). Thus, the positive Z scores indicate values above the mean, while negative Z scores values are below the mean in units of standard deviation. The scaled data is then converted into colors. Hierarchal clustering is based on the Euclidian similarity index. **(A)** Heat map of > 0.01% relative abundance at class level for Arabian Sea time series (ASTS). **(B)** Heat map of > 0.01% relative abundance at class level for India’s Idea 2 (II2)**. (C)** Heat map of > 0.01% relative abundance at class level for Bay of Bengal time series (BoBTS).

Hierarchical clustering of the heatmap, based on Euclidian similarity index at class level is represented in Figure 3. The clustering of bacterial communities at ASTS (Figure 3A) shows high relative abundance of γ-Proteobacteria and α-Proteobacteria. While the depths within the OMZ (150, 350, and 500 m) clustered together depicting similar bacterial communities, however at 40 m the bacterial communities differed from the other sampled depths. Most of the reads at class level were affiliated to the order Alteromonadales (γ-Proteobacteria) and SAR11 clade (α-Proteobacteria). At station II2 (Figure 3B) in the Arabian sea, the heatmap shows the clustering of surface depths (30 and 75 m) and the mid OMZ depths having low DO clustered together. SAR86 and SAR11 clade of the class γ-Proteobacteria and α-Proteobacteria respectively were dominant. The surface depths also had Oxyphotobacteria at high relative abundance, mostly contributed by the Synechococcales. While at BoBTS (Figure 3C), the most abundant bacteria belonged to α-Proteobacteria, δ- Proteobacteria, and γ-Proteobacteria with the clustering of mid-OMZ interphase (250 and 350 m) and the upper and lower OMZ interphase (150 and 530 m). The orders that were relatively abundant at these class were SAR11 clade (α-Proteobacteria), SAR324 clade (Marine group B) (γ-Proteobacteria), and Thiomicrospirales (δ- Proteobacteria).

The archaeal community at the three locations were dominated by the Euryarchaeota along the vertical depths. The AS locations (ASTS and II2) showed presence of other less dominant phyla viz., Crenarchaeota, Diapherotrites, Nanoarchaeota, and Thaumarchaeota. While at BoBTS location besides the Euryarchaeota, a less dominant Diapherotrites was recovered during analysis. The archaeal groups at class level showed a relatively high abundance of Thermoplasmata and Nitrososphaeria followed by less dominant Bathyarchaeia, Crenarchaeota *Incertae sedis*, Group 1.1c, Halobacteria, Marine Benthic Group A, Methanobacteria, Micrarchaeia, Thermococci, and Woesearchaeia (Supplementary Figure S3) at ASTS and II2 stations.

**FIGURE 4:**
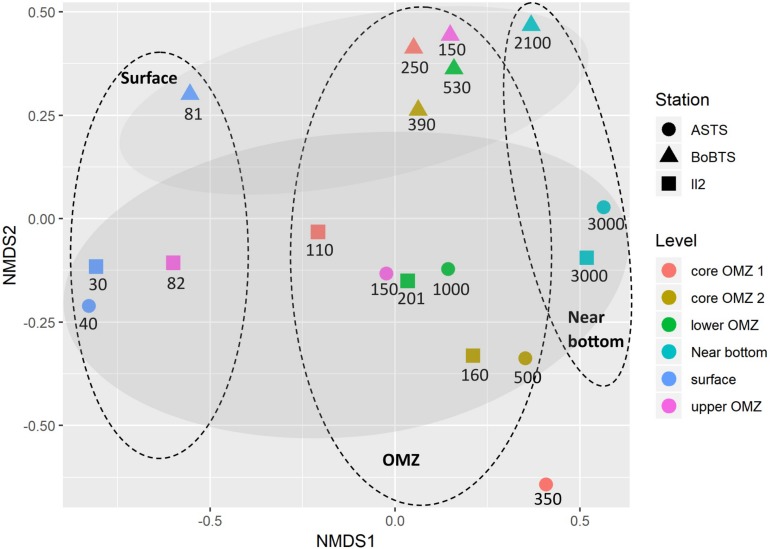
Non-metric multidimensional scaling (NMDS) plot depicting class-level bacterial composition from six depths of ASTS, BoBTS, and II2. Axis define 2D space that allows the best spatial representation of sample distance, based on Bray–Curtis distance with stress = 0.08. The depths in meters of each sample are mentioned below the respective symbol, while the oval dashes represent the three planes which divides the data based on vertical depth profile and the shaded oval regions represent grouping of data based on location.

Beta diversity separated the samples into abundance-weighted community composition, based on Bray–Curtis distance (stress = 0.08) (Figure 4) using NMDS ordination. The bacteria at class level grouped based on location and on vertical depth profile. Within the vertical depth profile, regardless of the sites the samples grouped into three planes; surface depths, the four OMZ depths and near bottom depths (Figure 4).

### Correlation With Environmental Parameters

**FIGURE 5:**
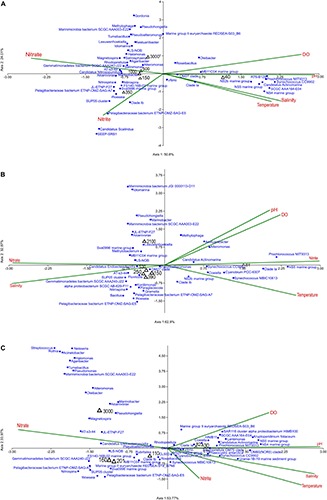
Canonical Correspondence Analysis ordination diagram of bacterial communities at generic-level at **(A)** Arabian Sea time series (ASTS), **(B)** Bay of Bengal time series (BoBTS), **(C)** India’s Idea 2 (II2) associated with environmental parameters. In red is the environmental parameters, black is depths sampled and blue is bacterial genera.

The effect of environmental conditions on prokaryotic community structure was studied at ASTS, BoBTS, and II2 sites along six depths. Identified genera along with six environmental variables [Temperature (°C), salinity (PSU), DO (μM), pH, NO_3_ (μM), and NO_2_ (μM)] were analyzed using CCA. For the ease of representation at each sampling site, only 0.1% and above relative abundance of genera were plotted in CCA excluding the unclassified reads. Results at ASTS showed that 74.81% of the community variation could be explained by the environmental parameters included in this study (Figure 5A). A triplot revealed the correlation between the physicochemical factors and genera *Nitrospina*, *Nitrospira*, *Woeseia*, SUP05 cluster, Pelagibacteraceae bacterium ETNP-OMZ-SAG-A7, Pelagibacteraceae bacterium ETNP-OMZ-SAG-E5, Clade lb, Sva0996 marine group, JL-ETNP-F27, FS140-16B-02 marine group, SEEP-SRB1 and *Candidatus* Scalindua were higher at 350 m depth, and were negatively correlated with DO and positively correlated with NO_2_. Of all the environmental factors analyzed, DO, nitrate and pH contributed significantly to the community variance at ASTS. At BoBTS, the mid depths (150, 250, 390, and 530 m) were negatively correlated to DO, NO_2_ and pH, while positively correlated to salinity (Figure 5B). Under low DO, NO_2_ and pH conditions, the abundant genera/OTUs present were Pelagibacteraceae bacterium ETNP-OMZ-SAG-A7, Pelagibacteraceae bacterium ETNP-OMZ-SAG-E5, SUP05 cluster, *Bacillus*, *Nitrospina*, *Fluviicola, Kordiimonas, Woeseia, Gramella Paraglaciecola*, and *Candidatus* Endoecteinascidia. The surface depth showed an abundance of *Synechococcus* CC9902, *Synechococcus* MBIC10613 and *Prochlorococcus* MIT9313 accompanied with high DO, NO_2_, and temperature conditions. Figure 5C shows the CCA plot of II2 location similar to ASTS and BoBTS location with mid-depths of 110, 160, and 201 m showing lower DO values, and harbored genera/OTUs viz; *Nitrospina, Pseudoalteromonas, Rubritalea, Roseibacillus, Woeseia*, SUP05 cluster, Pelagibacteraceae bacterium ETNP-OMZ-SAG-E5, Pelagibacteraceae bacterium ETNP-OMZ-SAG-A7, Sva0996 marine group, Clade lb, Marine group II euryarchaeote REDSEA-S19_B7N8 and Gemmatimonadetes bacterium SCGC AAA240-J22. The surface depths 30 m and 82 m showed relatively high abundance of *Synechococcus* CC9902, *Prochlorococcus* MIT9313, *Coxiella*, *Vibrio*, and *Candidatus* Actinomarina.

### Predictive Functional Studies

The results obtained showed high levels of genetic diversity, with organisms involved in various essential processes, such as metabolism, environmental, genetic information processing, cellular processes along with a few uncharacterized proteins. In general, peptide/nickel transport system substrate-binding protein (K02035), ribonuclease E (K08300), ATP-binding cassette, subfamily B, bacterial (K06147), 3-oxoacyl-[acyl-carrier protein] reductase (K00059), and methyl-accepting chemotaxis protein (K03406) were some of the few highly abundant genes at all three sites analyzed by Tax4Fun (Supplementary Figures S4a–c). The bacterial communities were predicted to harbor genes involved nitrogen and sulfur cycles (Supplementary Figures S5a,b). Further, analyzed results of nitrogen and sulfur metabolism across the sampled depths at the three stations was significantly different with *p* = 0.00966 (Supplementary Figure S6).

## Discussion

### Variation in Physicochemical Parameters

The DO concentration among the three sampled locations is consistent with results previously described in the Indian Ocean OMZs (Bristow et al., 2017; Mulla et al., 2017; Bandekar et al., 2018), where ASTS exhibited intense oxygen minima conditions at mid-depths than the Bay of Bengal (BoBTS), and II2 having oxygen values above the defined threshold (<20 μM). Salinity at BoBTS was lower in surface in comparison to ASTS and II2. Higher volumes of river run-off in BoB (1.6 × 10^12^ m^–3^ yr^–1^) compared to AS (0.3 × 10^12^ m^–3^ yr^–1^), and excessive evaporation over precipitation in AS, leads to surface waters being 3–7 PSU less saline in the BoB (Prasanna Kumar et al., 2002). Nitrate was detected in minute quantities in the surface waters across the sampled locations. The source of nitrate availability in the surface waters is governed by water mixing, diffusion of nitrate-rich deep ocean reservoir, and rate of biological production. Since AS and BoB are the two most productive regions in the Indian Ocean, this accounts for low surface nitrate concentrations (Qasim, 1999). While bacterial decomposition replenishes nitrate below the euphotic zone thus increasing nitrate concentration in mid and deeper depths (Zehr and Ward, 2002). Higher nitrite values observed at low DO make the environment conducive for biological processes such as denitrification. Such conditions of high nitrite accompanied with low DO were observed at ASTS (350 m) suggesting occurrence bacterial community governing such biological processes (De Sousa et al., 1996). While high nitrite concentrations in the surface waters drive nitrification process (De Sousa et al., 1996), which could be observed at II2 and BoBTS.

### Alpha Diversity of Bacterial Communities

Higher OTU richness and diversity (H) was observed at suboxic transition zones and near bottom, while it declined at the surface and within the core OMZ (DO < 3 μM) waters. Similar analysis of seasonal OMZs of Eastern Tropical South Pacific (ETSP) and Saanich Inlet showed 16S rRNA sequence richness declined within the OMZ (Zaikova et al., 2010; Bryant et al., 2012). In contrast, sequencing results of PCR amplified V3 16S rRNA gene and 16S rRNA gene clone library revealed that OTU diversity was higher in the AS core OMZ than the upper OMZ and surface waters (Jain et al., 2014; Bandekar et al., 2018). High bacterial diversity in the core OMZ has also been recorded at ETSP (Stevens and Ulloa, 2008). Recently, similar to the present study in the BoBTS, V3 hyper-variable region of 16S rRNA genes was sequenced (97% sequence similarity), reported high diversity at the core and lower OMZ than the surface. Due to restricted sampled depths and higher DO concentration within the core OMZ (1.5 μM) than the present study (0.6 μM) (Fernandes et al., 2019), this could be a reason for such divergent findings. Bryant et al. (2012) speculated lower redox potentials and less readily available organic matter conditions in the core OMZ of ETSP to be one of the reasons of low energy availability and thus the cause of reduced microbial richness. A comparison was done between metagenomic techniques employed and the total volume of water sampled at ASTS and BoBTS based on prior reports (Table 2). It was observed that in the present study higher OTU richness could be recovered at ASTS with the use of high throughput sequencing. However, at BoBTS, higher OTU richness was reported in Fernandes et al. (2019) where similar molecular identification method was employed. Notably, the DO concentration observed during Fernandes et al. (2019) study was higher than those observed in BoBTS-OMZ in the present study. This could explain lower OTU richness associated with decreased DO concentrations (Bryant et al., 2012).

**TABLE 2:** A comparative representation of metagenomic techniques used to obtain observed OTUs in the water column from ASTS and BoBTS OMZ locations.

**Location**	**Quantity of water sampled (L)**	**Technique used**	**Seasons sampled**	**Number of OTUs observed at sampled depths**						**Reference**
					
				**Surface**	**OMZ**				**Near bottom**	
						
				**0.5 m**	**43/50 m**	**250 m**	**500 m**	**1000 m**	**–**	
Arabian Sea, ASTS (17.0021° N, 67.9962° E)	2.5	DGGE	SIM^a^	8	10	6	6	7		Jain et al., 2014
			FIM^b^	7	8	7	5	5		
			NEM^c^	9	9	7	6	4		

				**5 m**	**DCM^d^**	**250 m**	**500 m**	**1000 m**	**–**	

Arabian Sea, ASTS (17.0021° N, 67.9962° E)	2.5	16S rRNA gene clones	SIM^a^	7	7	12	14	11		Bandekar et al., 2018
			FIM^b^	7	9	8	9	11		
			NEM^c^	5	7	11	12	12		

				**43 m**	**–**	**200 m**	**–**	**1000 m**	**–**	

Bay of Bengal, BoBTS (18.0027° N, 89.0174° E)	5	Illumina NextSeq V3 hypervariable amplicons	One-time sampling	2547		3431		4103		Fernandes et al., 2019

				**40 m**	**150 m**	**350 m**	**500 m**	**1000 m**	**3000 m**	

Arabian Sea, ASTS (16.997341° N, 68.005768° E)	10	Illumina Miseq V3–V4 hypervariable amplicons	One-time sampling	1347	1792	1131	1328	1646	1848	Present study

				**30 m**	**82 m**	**110 m**	**160 m**	**201 m**	**3000 m**	

Arabian Sea, II2 (9.000113° N, 67.999901° E)	10	Illumina Miseq V3–V4 hypervariable amplicons	One-time sampling	1249	1692	1560	1427	1457	1808	Present study

				**81 m**	**150 m**	**250 m**	**390 m**	**530 m**	**2100 m**	

Bay of Bengal, BoBTS (18.0027° N, 89.0174_° E)	10	Illumina NextSeq 500 V3 hypervariable amplicons	One-time sampling	1881	3233	1816	2256	2720	3298	Present study

*^*a*^Spring intermonsoon (SIM), ^*b*^fall intermonsoon (FIM), ^*c*^northeast monsoon (NEM), ^*d*^deep chlorophyll max.*

### Prokaryotic Community Structure

Prokaryotic community structure at ASTS, BoBTS, and II2 was found to be dominated by Proteobacteria. Proteobacteria and other dominant phyla/OTUs of Actinobacteria, Bacteriodetes, Cyanobacteria, Chloroflexi and Marinimicrobia identified in this study, were similar to those identified from a variety of pelagic OMZs (Ulloa et al., 2013; Fernandes et al., 2019). The Bacteroidetes was found to be abundant at surface waters and is known to be associated with phytoplankton (Fandino et al., 2001). Thus, it seems likely that in ASTS, BoBTS and II2, Bacteroidetes was observed at higher relative abundance in surface waters than in lower depths. Higher abundance of sequences from the anammox-group of *Candidatus* Scalindua was also identified at ASTS and II2 in AS. Even though the DO concentrations at BoBTS were lower than II2, there seem to be other physicochemical parameters such as nitrite concentrations in the water column that favor the proliferation of anammox-group in the OMZs. Absence of similar favorable conditions at BoBTS doesn’t allow anammox-group of bacteria to thrive at this location. The observed distribution of prokaryotes at each location along six depths, based on the taxonomic assignment at class level, was represented by hierarchical clustering analysis of the bacterial community. Considering Euclidian similarity index, a hierarchical clustering analysis showed a clear difference between samples from the surface, confirming dissimilar community structure in the surface waters than the bottom sampled depths. A similar observation was recorded within the ETSP OMZ, i.e., microbial communities from different depths within the OMZ clustered together, but those from surface waters differed (Bryant et al., 2012; Stewart et al., 2012). The γ-Proteobacteria and α-Proteobacteria were dominant at the sampled stations. Group of gammaproteobacterial sulfur oxidizers (GSO), SUP05 clade was relatively abundant at all three sampled sites under low DO (Figures 5A–C). At ASTS, the members of SUP05 were higher at the core OMZ (350 m) where the DO was lowest accompanied by high nitrite concentration. Due to their participation in various reactions such as oxidation of reduced sulfur compounds, reduction of oxidized nitrogen and CO_2_ fixation, SUP05 members play a significant role in major biogeochemical cycles in OMZs (Canfield et al., 2010). The major member affiliated to α-Proteobacteria was SAR11 clade. This group is also reported from Eastern Tropical North Pacific off Mexico OMZ participating in nitrate reduction (Tsementzi et al., 2016).

Most archaeal studies from the Arabian Sea and Bay of Bengal water column include reports on ammonia-oxidizing community (Pitcher et al., 2011; Wang et al., 2017) with a few studies highlighting the presence of archaeal community in the north Indian Ocean OMZ (Bandekar et al., 2018; Fernandes et al., 2019). Reports from off Costa Rica-OMZ and ETSP-OMZ indicate both the ammonia oxidizing archaea (Molina et al., 2010) and total archaeal community (Belmar et al., 2011) have high diversity at the core of the OMZ (Xia et al., 2017). Archaeal diversity from this study showed a similar trend of higher diversity at the core of the OMZ along all three sampled sites (ASTS- 350 m, II2- 160 m, and BoBTS- 250 m), when compared to the surface and near bottom oxic zones (Supplementary Table S1). Füssel (2014) had suggested that the co-occurrence of aerobic and anaerobic processes in the oxygen-depleted depths of the water column may explain the high archaeal diversity at these zones. The Euryarchaeota was recovered from all three stations in the present study, this phylum was reported to be present throughout the water column of ETSP and AS-OMZ (Belmar et al., 2011; Bandekar et al., 2018). In contrast to prior reports in the Indian Ocean OMZ, in the current study with the help of high throughput sequencing it was possible to recover fewer dominant phyla viz., Crenarchaeota, Diapherotrites, Nanoarchaeota, and Thaumarchaeota.

Multivariant analysis based on Bray–Curtis distance and NMDS ordination at class level grouped based on location separating Bay of Bengal sampling location (BoBTS) and Arabian sea sampling locations (ASTS and II2). This result must be interpreted with caution due to the influence of different hypervariable regions amplified at BoB-OMZ (V3) and AS-OMZ (V3–V4) in the present study. Recently, Kerrigan et al. (2019) reported taxonomic richness is linked toward the choice of hypervariable region selection and varies greatly between two different hypervariable regions generated from the same location. However, considering that the distance measure used not only takes taxa abundance into consideration but also relies on presence/absence, this limitation could be overlooked for further interpretation. Further, the implication of the use of different hypervariable regions does not impair class-level community composition and relative patterns of diversity (Kerrigan et al., 2019). This was also evident in the present study where hierarchical clustering at each location formed groups of surfaces, OMZ and near bottom depths which was also observed between sites. Thus, showing depth-wise bacterial similarity between ASTS, II2, and BoBTS. This vertical partitioning of bacterial communities between surface, OMZ and near bottom depths have been observed previously in the world OMZs (Stevens and Ulloa, 2008; Podlaska et al., 2012; Bandekar et al., 2018). The core of the ASTS (350 m) which deviate from the OMZ group thus indicating unique bacterial community at low DO and high nitrite conditions.

### Physicochemical Drivers of Prokaryotic Community Distribution

At the sampled stations among the six environmental parameters, DO, pH and temperature were consistent in contributing to the variation in the prokaryotic community, between the surface waters, OMZ and near bottom depths. The core OMZ (350 m) at ASTS showing a positive correlation toward nitrite and harbored relatively abundant genera involved in nitrate reduction (Pelagibacteraceae bacterium ETNP-OMZ-SAG-A7, Pelagibacteraceae bacterium ETNP-OMZ-SAG-E5), anammox (*Candidatus* Scalindua) and nitrite oxidation (*Nitrospina* and *Nitrospira*) processes. Similarly, previous studies have shown the presence of these genera in the permanent OMZs of Eastern Tropical North Pacific (ETNP), ETSP, AS, and BoB (Tsementzi et al., 2016; Bristow et al., 2017; Bertagnolli and Stewart, 2018; Fernandes et al., 2019). Archaeon marine Thaumarchaeota, *Candidatus* Nitrosopumilus was abundant at upper OMZ and near bottom depth (150 and 1000 m), this genus is a potential contributor of Archaea to global nitrogen and carbon cycles. *Candidatus* Nitrosopumilus is known to oxidize ammonium to nitrite and was also observed at ETSP, and Baltic Sea OMZ (Stewart et al., 2012). At mid-OMZ depths, prokaryotic community of BoBTS was negatively correlated to DO, nitrite and pH while showed positively correlated to salinity. Salinity is reported to act as a barrier in separating bacterial community having a substantial effect on bacterial community structure (Zhang et al., 2018). The relatively abundant genera indicated to be present at low DO and play a role in the nitrogen cycle. For example; *Bacillus* and *Nitrospina* are actively involved in nitrate reduction and nitrite oxidation respectively at low DO in the OMZs (Mulla et al., 2017; Bertagnolli and Stewart, 2018) while even genera such as *Kordiimonas* found to be involved in degradation of polycyclic hydrocarbons, aromatic and halogenated compounds making them inhabit these oligotrophic BoB waters. At II2 the depths 110, 160, and 201 m were negatively correlated with DO, inhabiting groups of bacteria involved in nitrate oxidation (LS-NOB, *Nitrospina*) and nitrate reduction processes (SUP05 clade, Pelagibacteraceae bacterium ETNP-OMZ-SAG-A7, Pelagibacteraceae bacterium ETNP-OMZ-SAG-E5). Previous studies have reported marine group II euryarchaeote REDSEA-S19_B1N8 was higher at the mid depths, and the Marine group II archaea would actively participate in critical processes of carbon and sulfur cycles which thrive at low oxygen concentrations (Zhang et al., 2015; Orsi et al., 2016). The surface depth of II2 at 82 m was positively correlated to nitrite with high relative abundance of *Synechococcus* MBIC10613. While at 30 m *Prochlorococcus* MIT9313 was influenced by high DO concentrations. It is reported that *Synechococcus* spp. can utilize nitrite as a sole nitrogen source, whereas most *Prochlorococcus* strains cannot grow on nitrite (Moore et al., 2002). Thus, it could be likely that *Synechococcus* was found to be positively regulated by nitrite than *Prochlorococcus.*

### Functional Genes From the Tax4Fun Prediction

The predictive metagenome approach by use of Tax4Fun provided insights into the functional capabilities of bacterial communities studied through 16S rRNA gene sequencing. Earlier studies with regards to functional gene studies are specific, dealing with selective marker genes (*nirS*, *nosZ*, *nifH*, *amoA*, *nxrB*) in the AS and BoB OMZ (Jayakumar et al., 2009; Bristow et al., 2017; Gomes et al., 2017; Wu et al., 2019). However, the use of Tax4Fun (in the present study) has highlighted the predictive functional gene processes capable by the bacterial communities present at ASTS, BoBTS and II2 locations in the OMZ. The results represented here showed a significant variation of nitrogen and sulfur metabolism at ASTS, BoBTS and II2, thus inferring different bacterial communities dominate and perform their functional processes at these sites. Based on these results, further investigation is required to understand the ecological roles of the dominant microbes present at the sampled OMZ locations.

## Conclusion

The OMZs harbor active bacterial community at low DO conditions, which play an active role in cycling of carbon, nitrogen, sulfur, and other elements. Due to steep oxygen gradients, the microbial communities switch between alternative electron acceptors and participate in many types of microbial metabolism. Thus, implementation of high-throughput amplicon sequencing techniques in gaining in depth microbial diversity are required to study the bacterial diversity in these chemically complex habitats. Results from this research study provided insights of diversity and distribution of bacteria in the OMZ water column using high throughput sequence-data. Higher bacterial richness was observed at ASTS, BoBTS, and II2 OMZs in the Indian Ocean, compared to previous studies reported from these regions. Even though the prokaryotic communities were similar to those described in a variety of pelagic marine OMZs, the physicochemical variations at each site provide perspective on determining the influence of abundant microbes and their niches. Further, the predictive functional analysis has highlighted that microbes in the OMZs may participate in a variety of metabolic processes other than the most significant nitrogen and sulfur pathways studied.

## Data Availability Statement

The datasets generated for this study can be found in the 16S rRNA gene sequence-data was submitted to the National Center for Biotechnology Information (NCBI) under BioProject ID PRJNA508851.

## Author Contributions

The work was conceived by the corresponding author and the experimental work was carried out by the first author as a part of her doctoral work. The result interpretation and manuscript writing were done by all the authors.

## Conflict of Interest

The authors declare that the research was conducted in the absence of any commercial or financial relationships that could be construed as a potential conflict of interest.
