# Supplementary material for: Diversity of Bacterial Community in the Oxygen Minimum Zones of Arabian Sea and Bay of Bengal as Deduced by Illumina Sequencing
Source: Front Microbiol. 2020 Jan 21;10:3153. doi: 10.3389/fmicb.2019.03153 (PMC6985565; doi:10.3389/fmicb.2019.03153)
Supplement: Supplementary file 1 [file Data_Sheet_1.doc]

**Fig. S1** Map showing sampling locations in Arabian Sea and Bay of Bengal OMZ


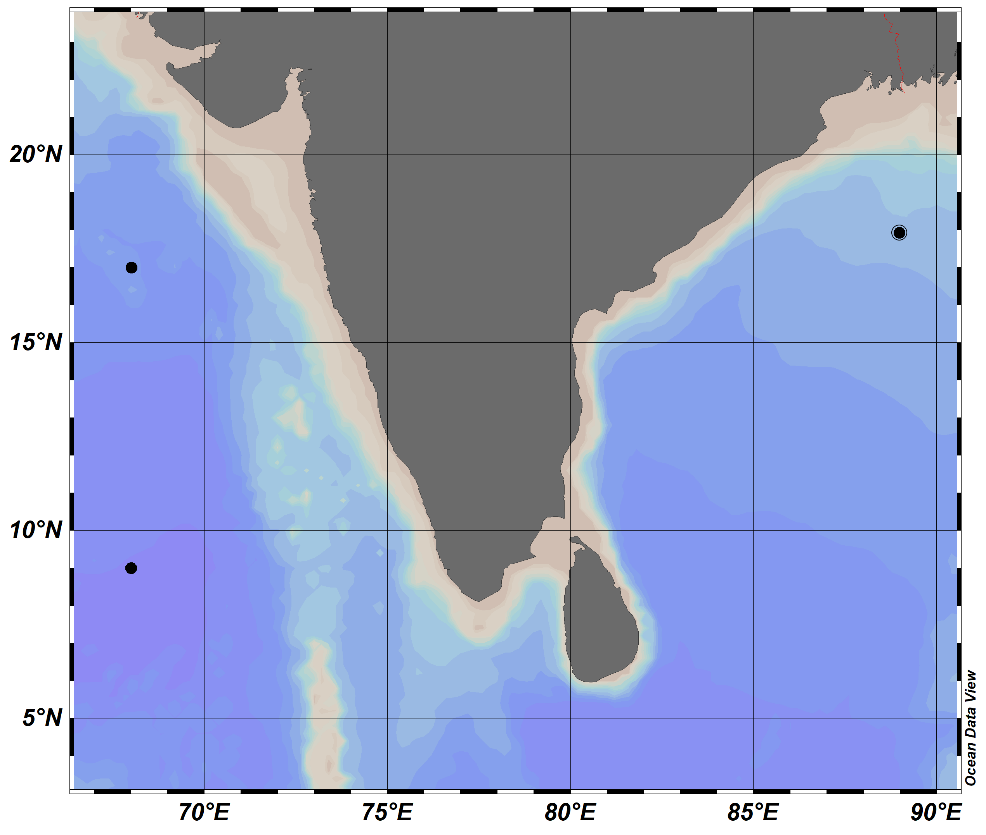


BoBTS

ASTS

*Arabian Sea*

II2

*Bay of Bengal*

**Fig. S2**. Rarefaction curves of ASTS, BoBTS and II2


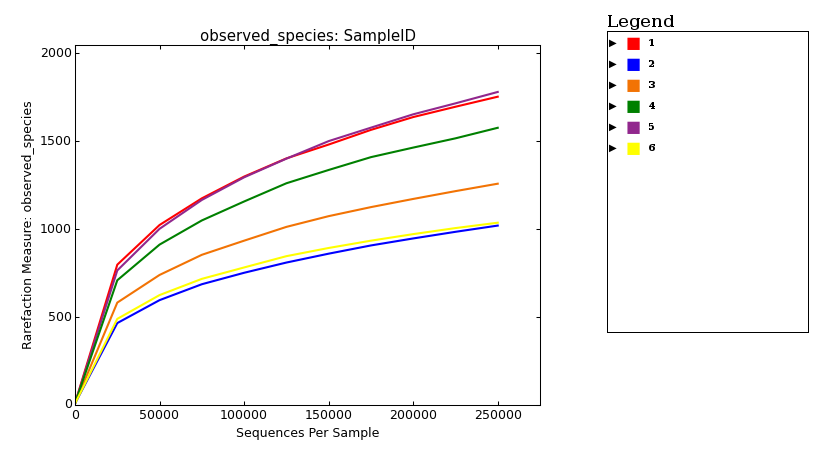


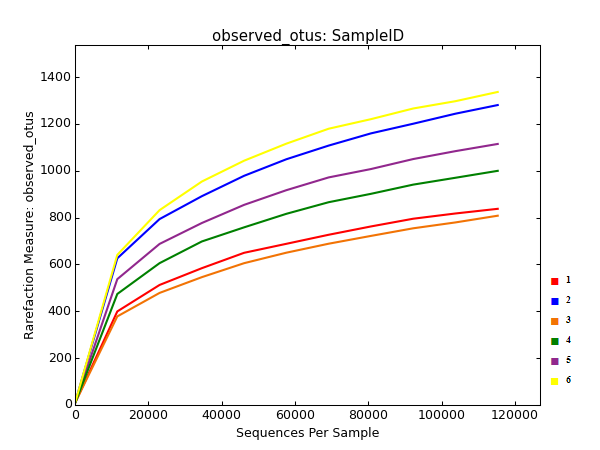


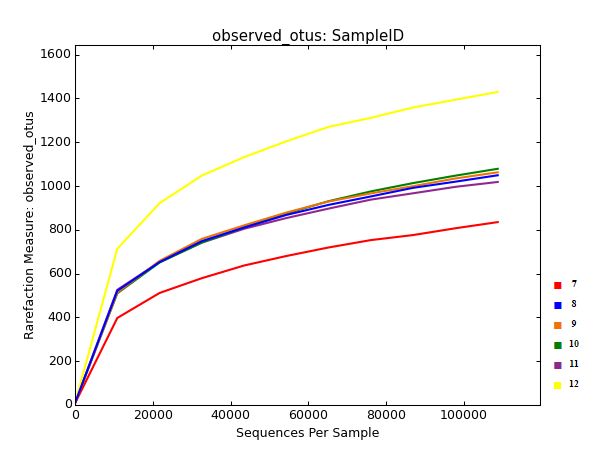


**Fig. S3** Archaea distribution (Class level) at ASTS, BoBTS and II2


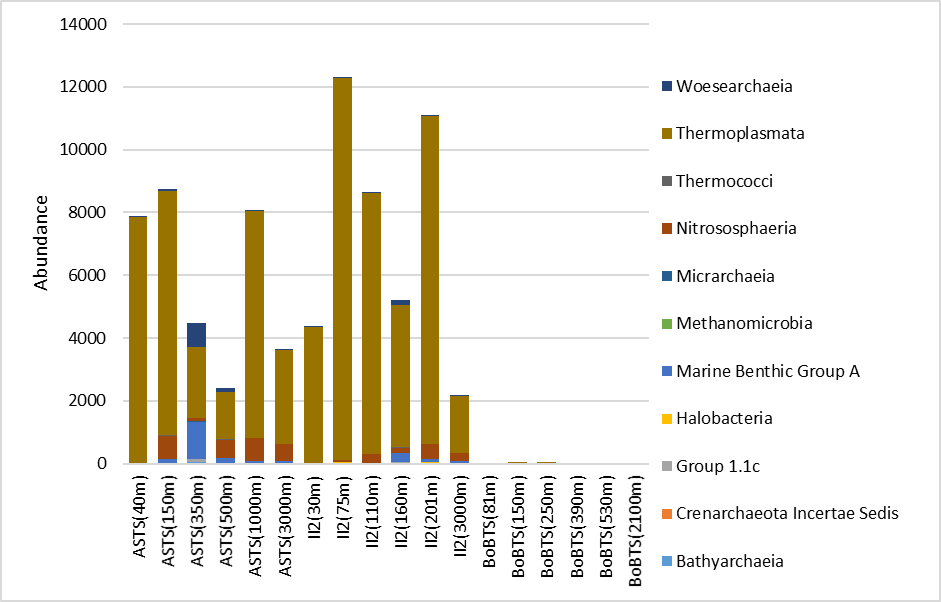


Fig S4a KEGG functional categories predicted from ASTS stations using Tax4Fun tool


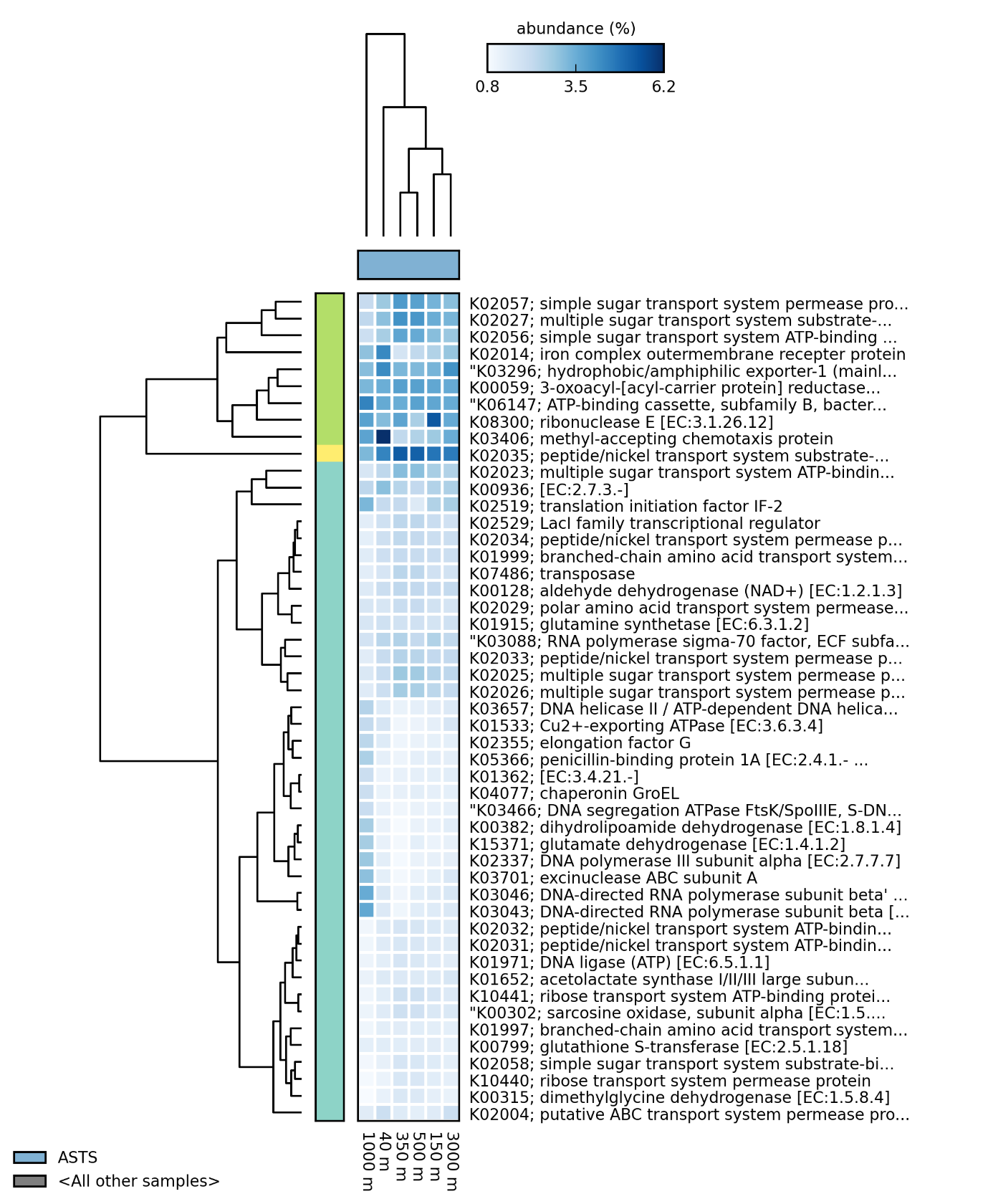


Fig S4b KEGG functional categories predicted from BoBTS stations using Tax4Fun tool


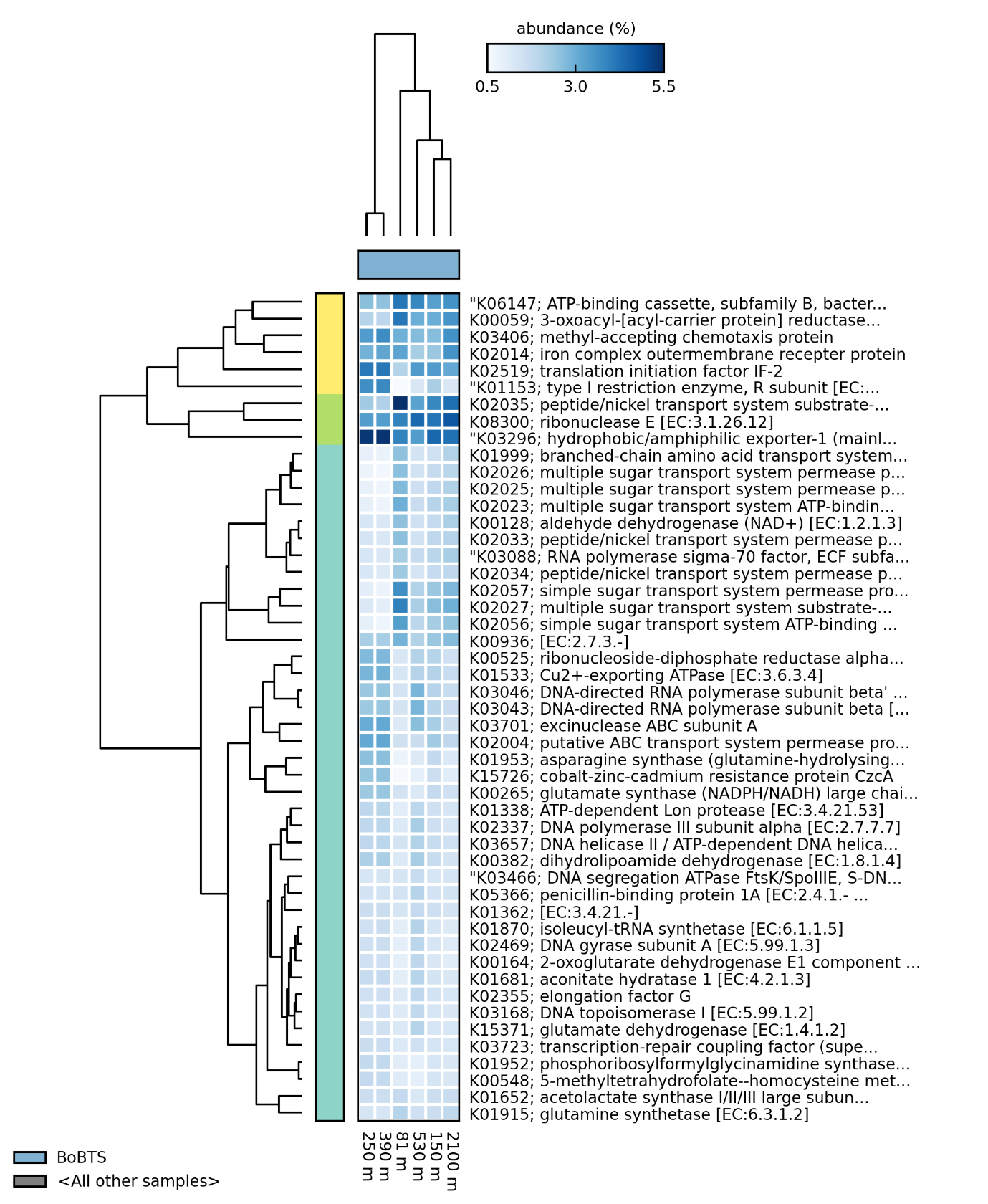


Fig S4c KEGG functional categories predicted from II2 stations using Tax4Fun tool


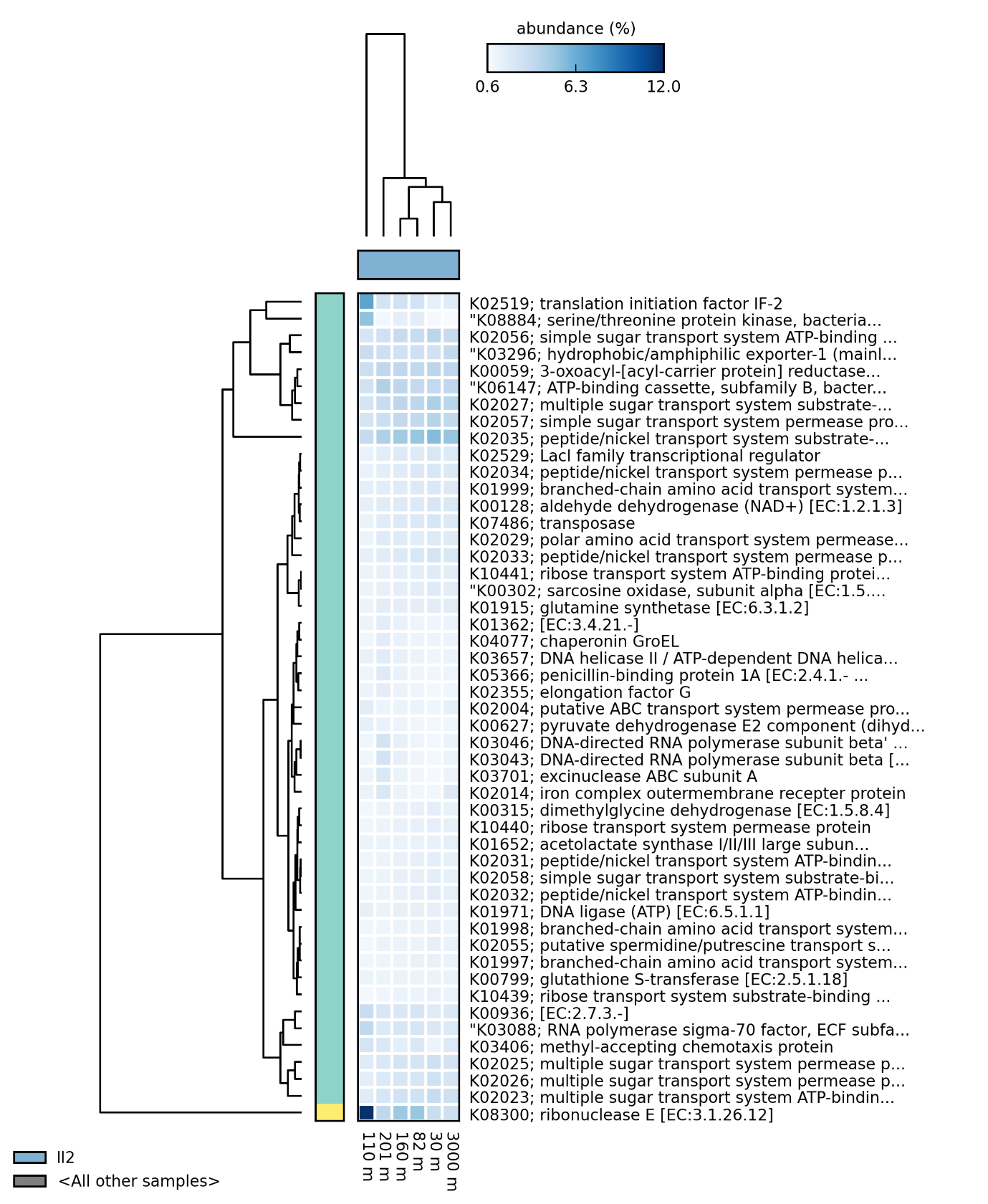


Fig S5a Nitrogen metabolism pathways at all 3 sampled sites


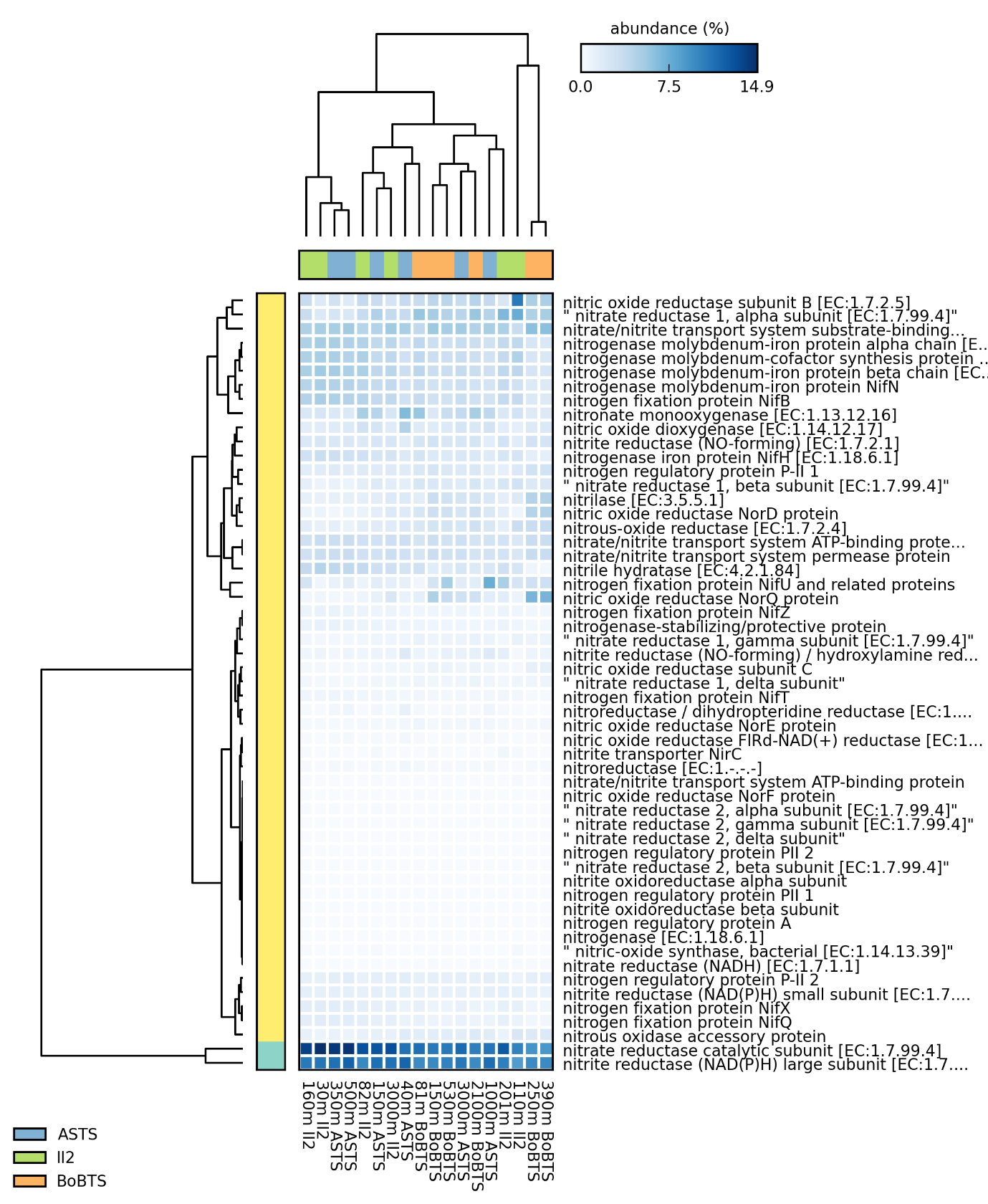


Fig S5b Sulphur metabolism pathways at all 3 sampled sites


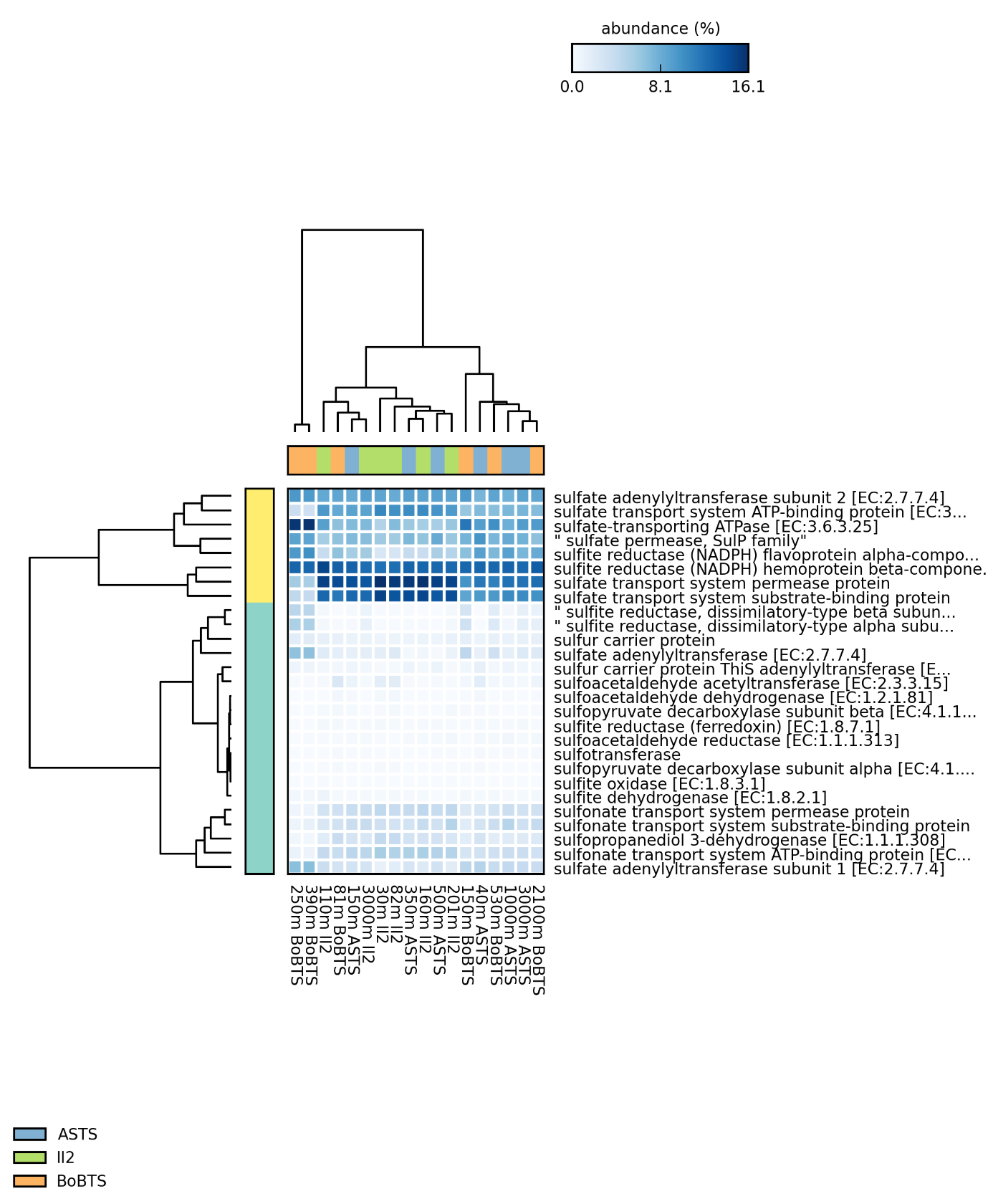


Fig. S6 Statistical analysis performed on KEGG pathways involved in nitrogen and sulphur metabolism


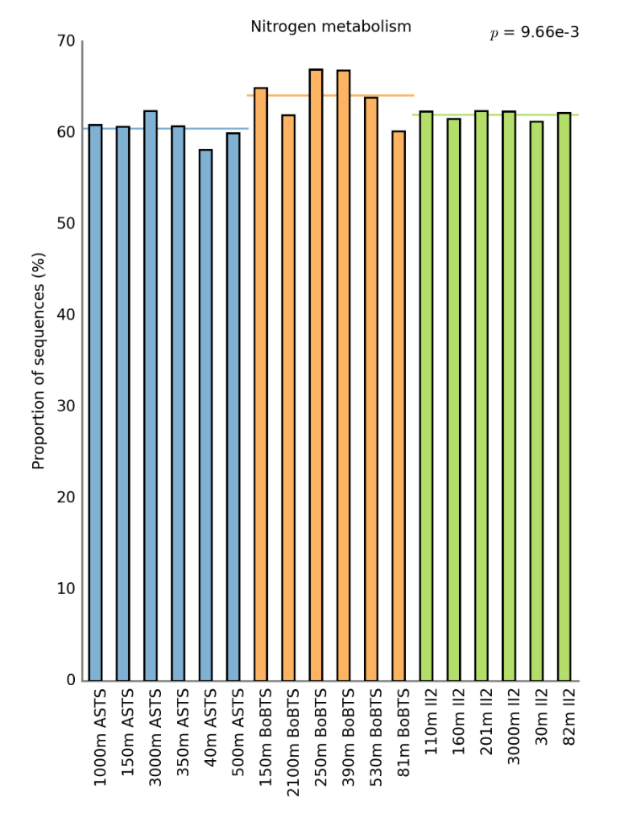

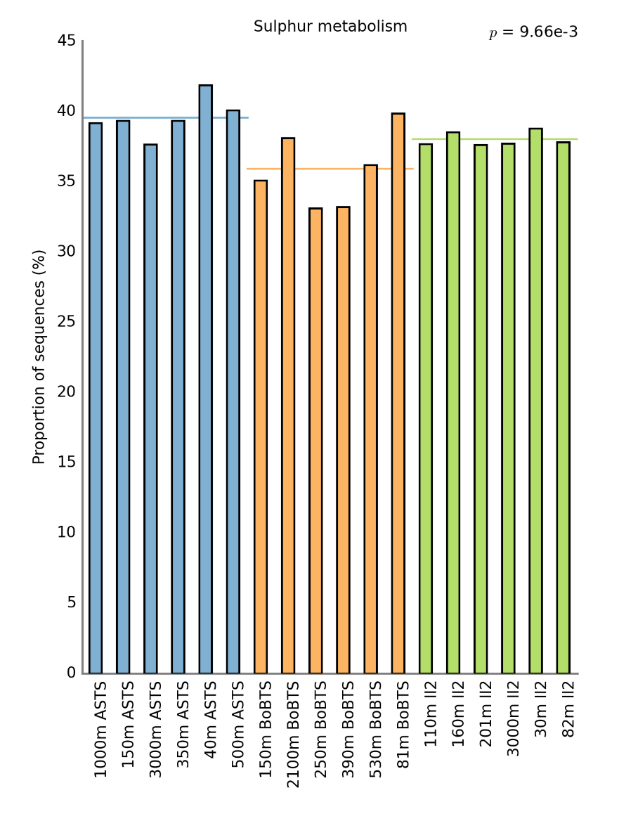


Table S1: Shannon_H diversity of archaeal community at class level along the sampled depths of ASTS, II2 and BoBTS

| ASTS | | II2 | | BoBTS | |
| --- | --- | --- | --- | --- | --- |
| 40 m | **0.01094** | 30 m | **0.02245** | 81 m | - |
| 150 m | **0.4336** | 82 m | **0.06904** | 150 m | **0.466** |
| 350 m | **1.274** | 110 m | **0.1651** | 250 m | **0.5623** |
| 500 m | **1.086** | 160 m | **0.5946** | 390 m | - |
| 1000 m | **0.3702** | 201 m | **0.2617** | 530m | - |
| 3000 m | **0.5613** | 3000 m | **0.5429** | 2100 m | - |
